# PFKFB4 Overexpression Facilitates Proliferation by Promoting the G1/S Transition and Is Associated with a Poor Prognosis in Triple-Negative Breast Cancer

**DOI:** 10.1155/2021/8824589

**Published:** 2021-06-09

**Authors:** Yu-Chen Cai, Hang Yang, Hong-Bo Shan, Hui-Fang Su, Wen-Qi Jiang, Yan-Xia Shi

**Affiliations:** ^1^Sun Yat-sen University Cancer Center, State Key Laboratory of Oncology in South China, Collaborative Innovation Center of Cancer Medicine, Guangzhou, Guangdong 510060, China; ^2^Department of Medical Oncology, Sun Yat-sen University Cancer Center, Guangzhou, Guangdong 510060, China; ^3^Department of Osteology, The First Affiliated Hospital of Zhengzhou University, Zhengzhou University, Zhengzhou, Henan 450000, China

## Abstract

**Background:**

6-Phosphofructo-2-kinase/fructose-2,6-biphosphate-4 (PFKFB4) is a key factor that plays an important role in tumorigenesis. However, its role in triple-negative breast cancer (TNBC) progression needs to be further validated. We investigated whether PFKFB4 is directly involved in the oncogenic signaling networks of TNBC.

**Methods:**

First, we assessed the expression level of PFKFB4 in tumor tissue specimens by immunohistochemistry and evaluated its prognostic value. Next, the effect of PFKFB4 on TNBC cell growth and associated mechanisms were investigated. Finally, the results were further verified in vivo.

**Results:**

We found that PFKFB4 overexpression was associated with an unfavorable prognosis in TNBC patients. PFKFB4 was overexpressed in TNBC cell lines in hypoxic environments, and its overexpression promoted tumor progression in vitro and in vivo. Further analyses demonstrated that the possible mechanism might be that PFKFB4 overexpression facilitates TNBC progression by enhancing the G1/S phase transition by increasing the protein level of CDK6 and phosphorylation of Rb.

**Conclusions:**

These data suggest that PFKFB4 plays significant roles in the tumorigenesis and development of TNBC.

## 1. Introduction

Breast cancer is a global disease and one of the main causes of female morbidity and mortality [1, 2]. Approximately 15% of breast cancers are defined as triple-negative breast cancer (TNBC), which lacks expression of the estrogen receptor (ER) and progesterone receptor (PR) and lacks overexpression of human epidermal growth factor 2 (HER2) [3, 4]. TNBC is one of the most aggressive breast cancer subtypes, and patients with TNBC have a worse clinical outcome than those with other breast cancer subtypes [4]. The development of metastasis in TNBC represents a highly complex and poorly understood process. Further research on TNBC is urgently required to advance our treatment approaches.

The most common feature of all cancer cells is the production of large amounts of lactate and pyruvate, which is due to enhanced glycolysis despite the presence of oxygen. This phenomenon was first described by Warburg [5]. Hypoxia and hypoxia-inducible factors (HIFs) are the master regulators of metastasis in solid tumors, which are usually exposed to a hypoxic state due to their indefinite growth and nutrient deficiency [6]. HIF-1 is a heterodimer with the HIF-1*β* subunit constitutively expressed and the HIF-1*α* subunit regulated in an oxygen-dependent manner. HIF-1*α* is known to be hyperactivated in TNBC [7, 8], while the mechanism and the target genes involved in the process by which HIF-1*α* regulates growth and metastasis in TNBC remain to be elucidated.

Hypoxia increases tumor glycolysis, angiogenesis, and cancer cell stemness, as well as invasion and metastasis. The activation of genes that increase the availability of oxygen, especially genes involved in glycolysis for maintaining cellular energy, is important in adaptations to hypoxia [9–11]. Glucose metabolism is regulated by fructose-2,6-bisphosphate, an allosteric activator of 6-phosphofructo-1-kinase [12, 13]. A single family of bifunctional 6-phosphofructo-2-kinase/fructose-2,6-biphosphate (PFK-2/FBPase-2 or PFKFB) enzymes is responsible for maintaining the cellular levels of fructose-2,6-bisphosphate [14], which has both kinase and phosphatase activities [15, 16]. Within a few years of the initial discovery of fructose-2,6-bisphosphate, multiple tissue-specific mammalian PFKFB isoenzymes were identified in several organs [17]. PFKFB isoenzymes are encoded by four different genes (PFKFB1-4) in cells and are considered key factors in many malignant conditions [18–20]. These genes encode proteins that differ not only in their tissue distribution but also in their function [14, 21]. PFKFB4 is located on chromosome 3 (bands p21-p22) and encodes an isoenzyme of PFKFB that was originally found in the testes [15]. Minchenko et al. suggested that PFKFB4 was induced by hypoxia in various cancer cell lines [19]. The overexpression of PFKFB4 in lung [22], breast, colon [18], and stomach cancer tissues [23] has been reported. Researchers have demonstrated the function of PFKFB4 in tumor growth by showing that silencing the gene inhibits the growth of human lung adenocarcinoma xenografts in athymic mice [24]. Independent studies have demonstrated that PFKFB4 is required for cancer cell survival [25, 26] but not for normal cell survival. This aberrant expression indicates that this protein may play a key role in tumor development. Hence, these studies demonstrated that PFKFB4 might be a useful molecular marker and potential target for the development of cancer therapeutics.

In the present work, to investigate the role of PFKFB4 in the progression of TNBC, we detected the expression of PFKFB4 in tissue specimens from TNBC patients by immunohistochemistry (IHC). Next, we studied the effects of PFKFB4 on cell proliferation and tumor growth and associated mechanisms in vitro and in vivo.

## 2. Materials and Methods

### 2.1. Patients and Tissue Specimens

A total of 180 TNBC patients treated between January 2006 and December 2015 with complete survival follow-up data and available tumor samples were enrolled in our study. All patients had undergone surgery at Sun Yat-Sen University Cancer Center, Guangzhou. The inclusion criteria were as follows: (1) patients underwent modified radical mastectomy; (2) the diagnosis of TNBC was confirmed by molecular biology and pathology; and (3) patients had complete follow-up information and pathological specimens available. Patients with incomplete records or with other malignant tumors at any time during the treatment and follow-up were excluded. All work was conducted in accordance with the Declaration of Helsinki. Written informed consent was obtained from every patient, and the study was approved by the Ethics Review Board of Sun Yat-Sen University Cancer Center. All patients were followed until August 2019.

### 2.2. Production of Tissue Microarrays (TMA), Immunohistochemistry, and Assessment

TMAs were constructed by extracting 2 mm diameter cores of histologically confirmed representative cancer cell areas from each original paraffin block according to a previously reported procedure [27]. Immunohistochemistry was performed on 4 mm sections of formalin-fixed, paraffin-embedded tissue samples that were dewaxed and rehydrated, and endogenous peroxidase activity was blocked with 0.3% H_2_O_2_ in methanol. The slides were boiled in 10 mM sodium citrate (0.05% Tween, pH 6.0) at high power for 10 min and medium power for 20 min in a microwave for antigen retrieval. A primary anti-PFKFB4 antibody (ab137785, 1 : 400, Abcam, Cambridge, MA, USA) was used and incubated overnight at 4°C. Then, a goat anti-rabbit secondary antibody was used at room temperature for 30 min. Finally, hematoxylin was used to counterstain nuclei. A negative control (NC) was obtained by omitting the primary antibody.

The specific intensities of staining were scored as follows: 0 = none; 1 = weak; 2 = moderate; and 3 = strong. The proportion scores were as follows: 0 = none; 1 = 1-10%; 2 = 11-50%; 3 = 51-80%; and 4 = >81% [28]. The scores for the percentage and intensity were multiplied to calculate an overall score, and the receiver operating characteristic (ROC) curve for the overall scores for PFKFB4 expression was plotted to select an appropriate cut-off score.

### 2.3. Cell Lines and Culture

The cell line used in this study was from the American Type Culture Collection and was identified by DNA (STR) profiling. MDA-MB-231 cells (a human breast cancer cell line, ER/PR/HER2 negative) were maintained in high-glucose (4.5 mg/ml) DMEM (Invitrogen, USA) supplemented with 10% fetal bovine serum (FBS; Gibco, USA). Cells were cultured under normoxia in a humidified atmosphere containing 5% CO_2_ at 37°C. For hypoxia, cells were exposed to an atmosphere composed of 5% CO_2_ balanced with N_2_ (<0.1% O_2_) in a controlled-oxygen MIC-101 chamber (Billups Rothenberg, Inc., Del Mar, CA, USA), and the duration of exposure was 4-6 h.

### 2.4. Construction of a Stable PFKFB4-Overexpressing Cell Line

A lentivirus carrying the pEZ-Lv105 plasmid encoding the full-length PFKFB4 ORF sequence (NM_004567.2) and an empty vector were purchased from GeneCopoeia. Each lentivirus was transfected separately into MDA-MB-231 cells in the presence of 5 *μ*g/ml polybrene (Sigma-Aldrich, USA). Puromycin (A1113803, Invitrogen; Thermo Fisher Scientific, Inc.) selection (10 *μ*g/ml) was started 24 h after transfection. Stable PFKFB4-overexpressing MDA-MB-231 cells (PFKFB4 cells) and empty vector cells (Vector cells) were established from isolated colonies and grown for subsequent assays. Untreated MDA-MB-231 cells were referred to as the NC group. The efficiency of PFKFB4 gene transfection was verified by Western blotting.

### 2.5. siRNA Silencing of PFKFB4 in MDA-MB-231 Cells

To knockdown the expression of PFKFB4, 231 cells were transfected with PFKFB4-specific siRNA or control siRNA (RiboBio, Co., Ltd., Guangzhou, China) using LipofectamineTM RNAiMAX transfection reagent (Invitrogen, Carlsbad, CA, USA) according to the manufacturer's instructions. The PFKFB4 siRNA sequence was sense: GGAAGGTCCTCAACGAGAT.

### 2.6. Cell Proliferation Assay

Tumor cells (1 × 10^3^/200 *μ*l/well) were plated in 96-well plates. After incubation for 24, 48, 72, or 96 h, a Cell Counting Kit-8 (CCK-8) solution was added into each well. The absorbance at 450 nm was measured by using a microplate reader (Bio-Rad, Hercules, CA, USA).

### 2.7. Colony Formation Assay

Cells were seeded in 6-well plates at a density of 100 cells per well and cultured for 14 days. Colonies were fixed with 4% paraformaldehyde (PFA) for 10 min and then stained with 0.1% crystal violet for 1 min. The cells were photographed and counted.

### 2.8. Cell Cycle Analysis

Cells were harvested, washed with PBS 3 times, and then, fixed in cold 70% ethanol at 4°C overnight. The cells were centrifuged at 500 g and washed in PBS twice. The cells were stained with 500 *μ*l propidium iodide (PI) solution (50 *μ*g/ml) containing 100 *μ*g/ml RNase A for 10 min in the dark and then analyzed by using a NovoCyte (ACEC Biosciences, Inc., USA).

### 2.9. Immunoblotting

Cells were lysed with cell lysis buffer (#9803, Cell Signaling Technology, Inc., USA), and equal amounts of cell lysates were electrophoretically separated using 8%, 10%, or 12% SDS-PAGE gels. The proteins were transferred to a PVDF membrane (Roche, USA). The membrane was blocked with 5% nonfat milk in Tris-buffered saline containing 0.1% Tween-20 for 1 h at room temperature and then incubated overnight at 4°C with antibodies. The following antibodies were used: anti-HIF-1*α* (#61959) from BD Transduction Laboratories (USA); anti-PFKFB4 (ab137785) from Abcam (USA); and anti-phosphor-Rb (Ser795) (#9301) and anti-CDK6 (#3136) from Cell Signaling Technology, Inc. (USA). Anti-beta-actin (#3700, Cell Signaling Technology, Inc., USA) was used as a loading control. HRP-conjugated anti-rabbit or anti-mouse (Santa Cruz) secondary antibodies were used. All blots were developed with 20× LumiGLO® Reagent and 20× peroxide (#7003, Cell Signaling Technology, Inc., USA).

### 2.10. Mouse Xenograft Model

In vivo experiments were performed using female nude mice (4- to 5-week-old) purchased from Beijing Vital River Laboratory Animal Technology Co., Ltd. The animals were kept in a temperature- and humidity-controlled facility with a 12-h light/dark cycle with free access to food. Animal experiments were approved by the Sun Yat-sen University Cancer Center ethics committee. To establish a breast cancer model, 1 × 10^5^ MDA-MB-231 cells (0.1 ml) were injected subcutaneously (five mice in each group). Every 3-4 days, tumor size was measured, and body weight was recorded. On the 39th day postinjection, the mice were sacrificed, and the tumors and organs were carefully removed, weighed, and subjected to immunohistochemical staining for Ki67 (ZA-0502, ZSGB-Bio, China) and PFKFB4 (ab137785, Abcam, USA). Tumor volumes were calculated with the following formula: *A* × *B*^2^/2 (*A*: the longest diameter; *B*: the diameter perpendicular to *A*).

### 2.11. Statistical Analysis

Statistical tests were carried out using SPSS version 19.0 (SPSS Inc., Chicago, IL, USA). Differences in mean values were evaluated using Student's *t*-test. Differences in frequencies were assessed with the chi-square test. Overall survival (OS) was calculated from the time of diagnosis to the day of death or the last date of follow-up. Disease-free survival (DFS) was defined as the duration between the time of disease diagnosis and the time of tumor relapse. Survival curves were plotted using the Kaplan-Meier method and compared with the log-rank test. Multivariate Cox proportional hazard models were used to define the potential prognostic significance of individual parameters. A ROC curve and the median were used to determine the cut-off value to distinguish high and low PFKFB4 expression. A two-sided *p* value less than 0.05 was considered significant.

## 3. Results

### 3.1. Relationships between PFKFB4 Expression and Clinicopathological Factors

**Figure 1:**
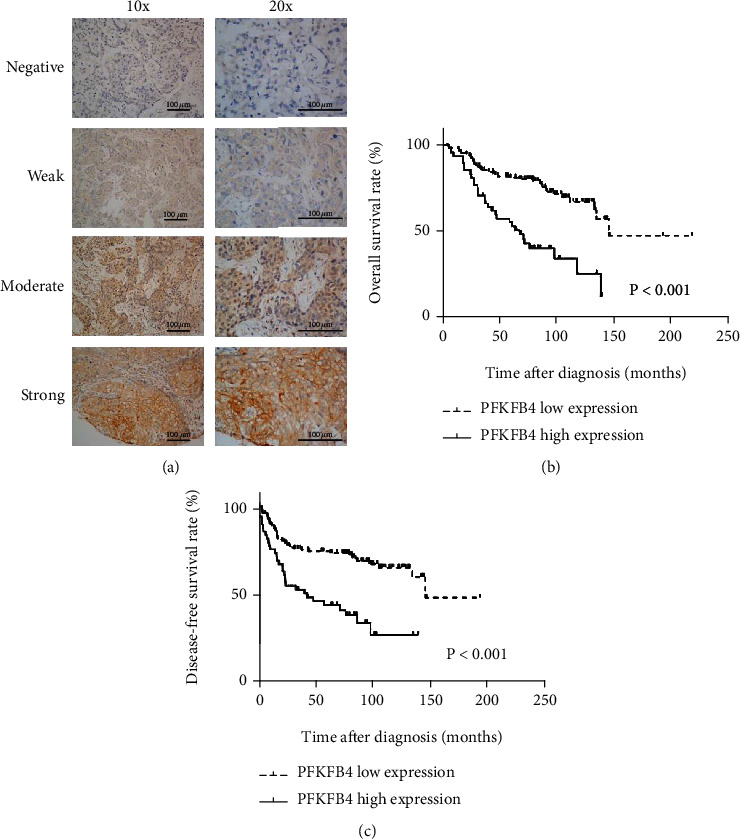
The expression of PFKFB4 in tumor tissue specimens and its prognostic value in TNBC patients. (a) Immunohistochemical analyses of PFKFB4 expression were performed with TNBC samples (scale bar: 100 *μ*m). The protein staining was mainly distributed in the cytoplasm. PFKFB4 overexpression was associated with relatively poor OS (b) and DFS (c, ^∗^*p* < 0.05) by survival curve analysis.

There were 180 female TNBC patients who were defined as having stage I to IV disease on the basis of the American Joint Committee on Cancer TNM staging manual (8^th^ edition updates), with a mean age of 50 years (range of 17-78 years), enrolled in our study. The assessment of PFKFB4 is shown in Figure 1. The area under the ROC curve was 0.657 (*p* < 0.001, 95% confidence interval (CI) 0.574-0.740). An overall score of 5 maximized the Youden Index (sensitivity (0.448) + specificity (0.832) − 1 = 0.280), indicating 5 to be the optimal cut-off score. Thus, expression of the PFKFB4 protein with a score of 0–4 was designated “low or weak expression” and that with a score of 6–12 was designated “high or strong expression.”

The PFKFB4 protein was located in the cytoplasm (Figure 1(a)), and the high-expression rate of TNBC tissues was 26.7%. We further found important relationships between high levels of PFKFB4 expression in TNBC and disease relapse (*p* < 0.001) and death rates (*p* < 0.001) (Table 1).

### 3.2. Prognostic Value of PFKFB4 Overexpression in TNBC Patients

As demonstrated in Figure 1(b), a significant difference in 5-year overall survival (OS) was observed between the high PFKFB4 expression group (mean survival = 76.4 months) and the low expression group (mean survival = 151.1 months, *p* < 0.001). Additionally, a statistically significant difference in 5-year disease-free survival (DFS) was observed when patients were stratified by PFKFB4 expression (Figure 1(c), *p* < 0.001). In univariate and multivariate survival analyses, a high PFKFB4 protein level was an important prognostic factor for shortened OS (*p* < 0.001) and DFS (*p* < 0.001) (Tables 2 and 3). Thus, our findings indicate that PFKFB4 overexpression is significantly associated with the prognosis of TNBC.

### 3.3. Expression Level of PFKFB4 and Its Effects on Cell Features of MDA-MB-231 Cells

**Figure 2:**
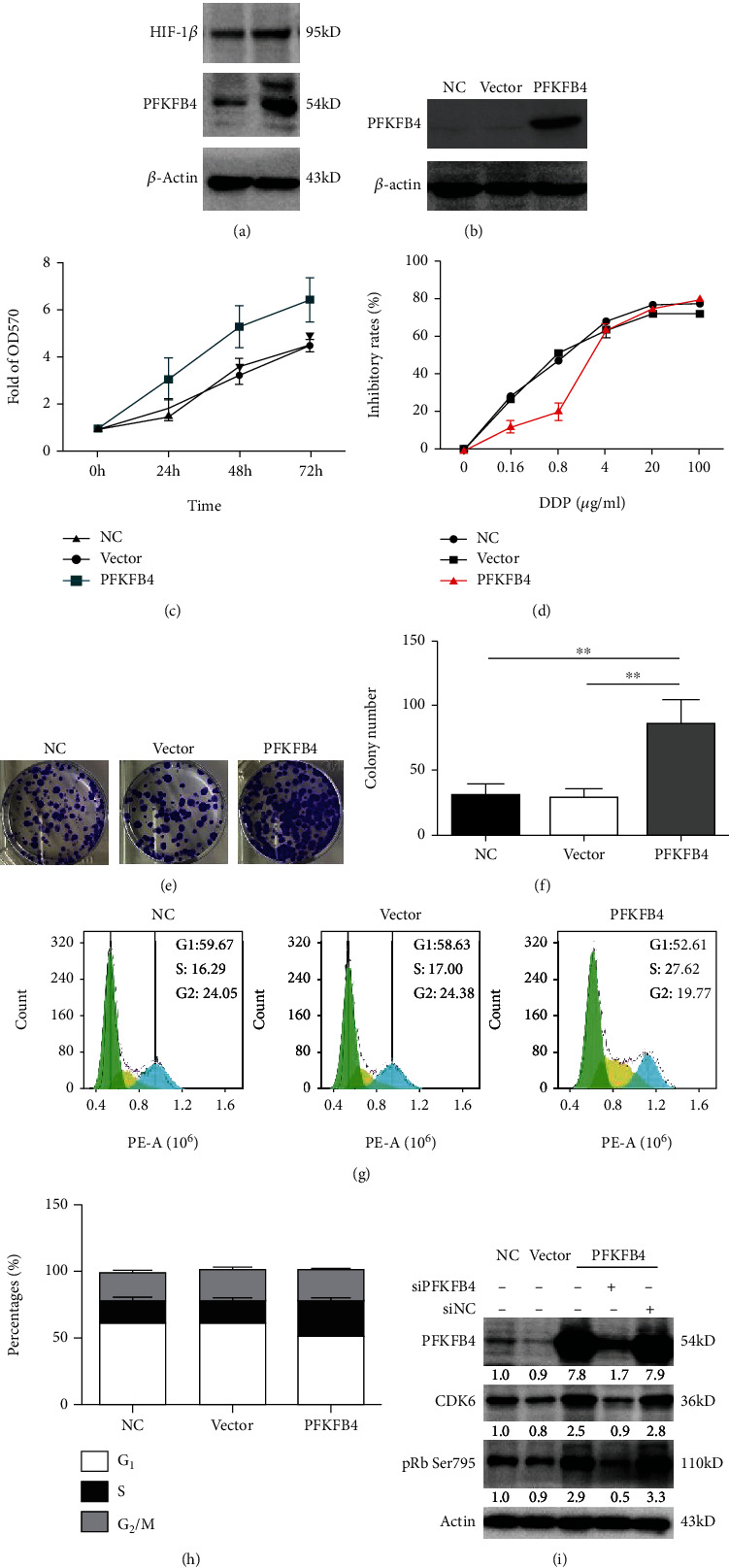
PFKFB4 overexpression facilitates the proliferation of breast cancer cells by promoting the G1/S phase transition. (a) A WB assay revealed that 231 cells had elevated levels of HIF-1*α* and PFKFB4 following hypoxia treatment. Stable PFKFB4 overexpression (b) promoted the growth of TNBC cancer cells (c) and these cells exhibited more resistance to cisplatin (d) than negative control (231 NC) and empty vector (Vector) cells. (e, f, ^∗^*p* < 0.05) Overexpression of PFKFB4 remarkably promoted the formation of cell clones compared with control expression. (g, h) Flow cytometric analysis found that PFKFB4 overexpression increased the percentage of S-phase cells. Each bar represents the mean of three independent experiments (^∗^*p* < 0.05). (i) A WB assay showed increases in CDK6 and pRb Ser795 expression, which hinted that PFKFB4 overexpression might promote TNBC cell proliferation by facilitating the G1/S phase transition.

The HIF-1*α* and PFKFB4 proteins were expressed at low levels under normal conditions in the MDA-MB-231 cell line.  Figure 2(a) demonstrates that the cell line displayed elevated levels of HIF-1*α* and PFKFB4 following treatment with hypoxia (*p* < 0.05). Stable 231 PFKFB4 and Vector cell lines were established and upregulation of PFKFB4 protein was confirmed by Western blotting (Figure 2(b)). The proliferative activity of 231 PFKFB4 cells increased (Figure 2(c)). Furthermore, we tested the inhibitory effect of DDP on three groups of cells. The IC_50_ of 231 PFKFB4 cells (2.92 ± 0.13 *μ*g/ml) was significantly higher than NC group (0.72 ± 0.02 *μ*g/ml, *p* < 0.001) and Vector group (0.69 ± 0.02 *μ*g/ml, *p* < 0.001), indicating that 231 PFKFB4 cells exhibited more resistance to cisplatin (Figure 2(d)). Additionally, we found that compared with control expression, PFKFB4 overexpression could remarkably promote the formation of cell clones (Figures 2(e) and 2(f), *p* < 0.05).

### 3.4. PFKFB4 Overexpression Facilitates the Proliferation of Breast Cancer Cells by Promoting the G1/S Phase Transition

To determine whether PFKFB4 is involved in the regulation of cell cycle progression, we saturated transfected MDA-MB-231 cells with PI, which stains the nuclear contents of a cell. Then, the cells were subjected to fluorescence-activated cell sorting (FACS). The findings showed that the percentage of cell number in S phase significantly increased in the 231 PFKFB4 group (25.8 ± 1.8%) (*p* < 0.01) compared with the 231 NC (16.1 ± 2.6%) and Vector groups (18.6 ± 1.7%) (Figures 2(g) and 2(h)). Furthermore, elevated levels of CDK6 and pRb Ser795, which are associated with the G1/S checkpoint, were observed in 231 PFKFB4 cells (Figure 2(i)). These combined results suggest that the PFKFB4 protein induces an increase in the frequency of cells in the S phase of the cell cycle in a TNBC cell line, resulting in the promotion of proliferation.

### 3.5. Overexpression of PFKFB4 Promoted Tumor Growth In Vivo

**Figure 3:**
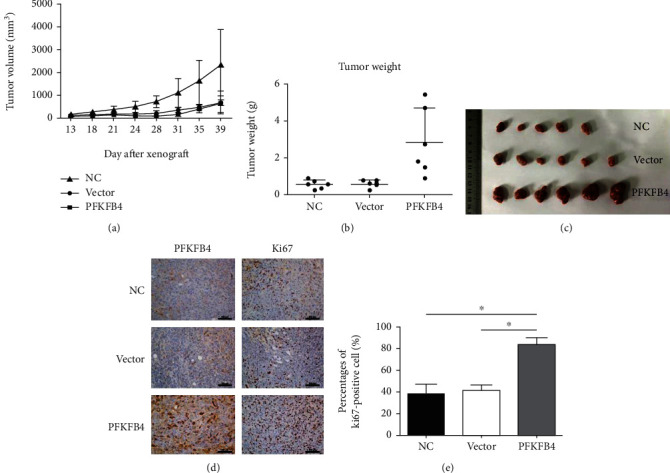
Overexpression of PFKFB4 promoted tumor growth in vivo. Both tumor volumes and weights were larger in the 231 PFKFB4 group (a–c) than in the control groups (^∗^*p* < 0.05). (d, e) Compared to the control groups, the 231 PFKFB4 group exhibited significantly more protein expression of Ki67 (^∗^*p* < 0.05).

We further explored the impact of PFKFB4 overexpression on the growth of MDA-MB-231 cells in vivo. 231 NC, Vector, or 231 PFKFB4 cells were injected subcutaneously into right back of nude mice. The NC, Vector, and 231 PFKFB4 cells formed tumors in the nude mice after injection. In day 28, the tumor volume in the 231 PFKFB4 group was 381.4 ± 135.7 mm^3^, which increased significantly comparing with the NC group (199.1 ± 47.7 mm^3^, *p* < 0.05) and the vector group (132.1 ± 64.3 mm^3^, *p* < 0.01) (Figures 3(a) and 3(c)). In day 35, the tumor volume in the 231 PFKFB4 group was 712.5 ± 253.7 mm^3^, which increased significantly comparing with the NC group (207.9 ± 102.0 mm^3^, *p* < 0.01) and the vector group (153.1 ± 62.7 mm^3^, *p* < 0.01). In day 46, the mice were and the tumors were weighed. The tumor weight in the 231 PFKFB4 group was 2.87 ± 1.85 g, which increased significantly comparing with the NC group (0.57 ± 0.23 g, *p* < 0.05) and the vector group (0.60 ± 0.21 g, *p* < 0.05) (Figures 3(b) and 3(c)). These results indicated that overexpression of PFKFB4 promoted tumor growth in vivo, which is consistent with the results from the in vitro experiments. To confirm the relationship between PFKFB4 overexpression and tumor proliferation, immunohistochemical staining for markers of proliferation was performed on tumor tissue samples. Compared to the control group, the PFKFB4 overexpression group exhibited more significant protein expression of Ki67 (Figures 3(d) and 3(e), *p* < 0.05).

## 4. Discussion

In human cells, bifunctional PFKFB family members control the steady-state cytoplasmic levels of fructose-2,6-bisphosphate, which activates the key enzyme (6-phosphofructo-1-kinase) in glycolysis [23]. PFKFB3 and PFKFB4 are the two primary isoenzymes overexpressed in various kinds of human cancers. PFKFB3 and PFKFB4 are widely involved in many biological processes, such as cell cycle regulation, autophagy, and apoptosis [29].

The results of this investigation indicate that the PFKFB4 protein is constitutively expressed in TNBC cells and that hypoxia significantly induces PFKFB4 expression in MDA-MB-231 cells. The regulation of expression appears to be related to a dependent mechanism that contains the activation of HIF-1 [30–32]. HIF-1 is a key factor that dominates the adaptation of cells to hypoxia and upregulates the expression of a series of genes involved in glycolysis. As shown in Figure 2(a), hypoxia increased the expression level of PFKFB4, and this phenomenon was correlated with an enhanced protein level of HIF-1*α*. These data suggest that hypoxic induction of PFKFB4 protein expression is mediated by HIF-1*α*. Since previous studies have demonstrated that the PFKFB4 gene is expressed in many tumor cell lines derived from different tissues [18, 19, 22], we provide consistent evidence that the testis isoform of the PFKFB protein is also expressed in TNBC cells.

Using multiple approaches, we demonstrated that (i) overexpression of PFKFB4 increased the proliferative ability of cancer cells; (ii) 231 cells expressing a high level of PFKFB4 exhibited increased resistance to cisplatin; and (iii) PFKFB4 expression at a high level promoted growth and invasion in vivo. Numerous studies have demonstrated the importance of PFKFB4 in cell malignancy [33, 34], and depletion of PFKFB4 was shown to inhibit tumor growth in a xenograft model [25]. Taken together, these data provide possible evidence that a significant function of PFKFB4 is promoting the growth of TNBC cells. The current research is the first study to demonstrate that the expression of PFKFB4 is significantly associated with prognosis in TNBC patients. Our results suggest that PFKFB4, at the protein level, has strong predictive value and is sufficient to predict the risks of recurrence and progression in TNBC. PFKFB4 may be an important therapeutic target for the prevention of progression and needs to be further explored. Previous studies demonstrate that PFKFB4 is overexpressed in breast cancer [18], promotes breast cancer cell stemness [35] and metastasis [36], and drives a protein signature that correlates with poor survival in patients [37]. We propose that PFKFB4 has important functions in TNBC progression and can serve as a useful independent prognostic marker in the clinical setting.

Considering the central role of DNA metabolism in the evolution of TNBC, we tested the effects of PFKFB4 overexpression on cell cycle progression. This study found that PFKFB4 regulated the cell cycle by regulating the G1/S phase transition. Cyclin D1 interacts with CDK4/6 to phosphorylate Rb (pRb), causing E2F to dissociate from the Rb-E2F complex, which is essential for DNA replication. Therefore, phosphorylating Rb allows the release of S phase-promoting transcription factors and is indicative of cell proliferation. The increase in the pRb level is essential for the G1/S phase transition [38, 39]. Palbociclib, an inhibitor of CDK4 and CDK6, was approved by the FDA in 2015 for the treatment of breast cancer. Because of the key role of CDK6 in PFKFB4-overexpressing tumors, palbociclib administration is considered an effective strategy for this type of cancer. In addition, Ki67, a protein marker of cell proliferation, is expressed only in the G1, S, and G2 phases, not in the G0 phase, and is a prognostic factor in breast cancer [40]. More Ki67 protein expression was observed in the PFKFB4 overexpression nude mouse group than in the control mouse groups in this study. For all of the reasons mentioned above, we propose that PFKFB4 overexpression facilitates the proliferation of TNBC cells by enhancing the G1/S transition by increasing the CDK6 level and phosphorylating Rb.

Although this is the first analysis to support the conclusion that the kinase activity of PFKFB4 is essential for TNBC progression, we discuss a specific mechanism that differs from the mechanisms identified in other studies. Previous studies identified that reduced lactate secretion and intracellular ATP levels were observed in malignant cells when PFKFB4 was silenced [26, 33], and the induction of lipid synthesis, which is required for cancer cell growth, was inhibited, possibly by reducing NADPH availability [41]. Moreover, other studies found that PFKFB4 might control cell survival via Akt signaling and regulate caspase 3 or 7 activity and the levels of reactive oxygen species [33, 34]. Minchenko et al. indicated that overexpression of PFKFB4 in malignant tumors correlated with enhanced expression of HIF-1a, glucose transporter 1 (Glut1), and vascular endothelial growth factor (VEGF), which are known HIF-1-dependent genes [18, 42]. Furthermore, in breast cancer, it was discovered that the enzyme PFKFB4 activates the transcriptional coactivator SRC-3 to drive the occurrence and development of tumors [37].

## 5. Conclusions

In summary, we demonstrated that PFKFB4 was overexpressed in TNBC cell lines in hypoxic environments; the overexpression of PFKFB4 promoted cell proliferation, clone formation, and drug resistance in vitro and tumorigenicity in vivo; and high PFKFB4 expression was significantly associated with a poor prognosis in TNBC patients. Furthermore, PFKFB4 overexpression might facilitate TNBC progression by enhancing the G1/S transition by increasing the CDK6 level and phosphorylating Rb. We believed that inhibiting PFKFB4 could be an effective strategy for TNBC treatment and that PFKFB4 suppression in combination with a CDK4/6 inhibitor needs further exploration.

## Figures and Tables

**Table 1:** Associations between the PFKFB4 expression and clinicopathologic factors of 180 patients with triple-negative breast cancer.

Characteristics	PFKFB4 expression (cases)		*p* value
	Low	High	
Total	132	48	
Age (years)			
≤30	6 (4.5%)	1 (2.1%)	0.450
>30	126 (95.5%)	47 (97.9%)	
Family history of cancer			0.761
No	122 (92.4%)	45 (93.8%)	
Yes	10 (7.6%)	3 (6.3%)	
Menopause			0.289
No	68 (51.5%)	29 (60.4%)	
Yes	64 (48.5%)	19 (39.6%)	
History of other neoplasms			0.907
No	127 (86.2%)	46 (95.8%)	
Yes	5 (3.8%)	2 (4.2%)	
Stage			0.537
0/I/II	89 (66.7%)	30 (74.7%)	
III	43 (33.3%)	18 (25.3%)	
T status			0.163
Tis/T1/T2	111 (84.1%)	36 (75.0%)	
T3/T4	21 (15.9%)	12 (25%)	
Lymph node metastasis			0.636
N0	63 (47.7%)	21 (43.8%)	
N1-3	69 (52.3%)	27 (56.2%)	
Vascular invasion			
No	107 (81.1%)	34 (70.8%)	0.141
Yes	25 (18.9%)	14 (29.2%)	
Disease recurrence			
No	89 (67.4%)	17 (35.4%)	**0.000**
Yes	43 (32.6%)	31 (64.6%)	
Death			
No	95 (72.0%)	18 (37.5%)	**0.000**
Yes	37 (28.0%)	30 (62.5%)	

**Table 2:** Univariate and multivariate analyses of the overall survival of the 180 TNBC patients. Stage III disease, lymph node metastasis, and high PFKFB4 expression were independent risk factors for a reduced OS rate (HR: hazard ratio; CI: confidence interval).

Variables	Overall survival			
	Univariate analysis		Multivariate analysis	
	HR (95% CI)	*p* value	HR (95% CI)	*p* value
Age ≤ 30 years old	0.877 (0.275-2.797)	0.824		
Family history of cancer	1.714 (0.781-3.762)	0.179		
Menstruating	1.280 (0.787-2.083)	0.320		
History of other neoplasms	1.384 (0.433-4.419)	0.584		
Stage III disease	4.400 (2.675-7.238)	**0.000**	2.739 (1.444-5.195)	**0.008**
T3/T4	3.138 (1.889-5.215)	**0.000**	1.320 (0.685-2.543)	0.407
Lymph node metastasis	3.714 (2.088-6.606)	**0.000**	2.336 (1.207-4.520)	**0.012**
Vascular invasion	3.107 (1.891-5.104)	**0.000**	1.351 (0.742-2.459)	0.325
High PFKFB4 expression	3.148 (1.928-5.139)	**0.000**	4.061 (2.424-6.831)	**0.000**

**Table 3:** Univariate and multivariate analyses of the disease-free survival of the 180 TNBC patients. Stage III disease, lymph node metastasis, and high PFKFB4 expression were independent risk factors for a reduced DFS rate (HR: hazard ratio; CI: confidence interval).

Variables	Disease-free survival			
	Univariate analysis		Multivariate analysis	
	HR (95% CI)	*p* value	HR (95% CI)	*p* value
Age ≤ 30 years old	0.916 (0.288-2.912)	0.881		
Family history of cancer	1.491 (0.683-3.259)	0.316		
Menstruating	1.409 (0.883-2.248)	0.151		
History of other neoplasms	1.264 (0.397-4.022)	0.691		
Stage III disease	4.112 (2.575-6.566)	**0.000**	2.494 (1.376-4.520)	**0.003**
T3/T4	3.037 (1.860-4.959)	**0.000**	1.443 (0.768-2.710)	0.255
Lymph node metastasis	3.426 (2.011-5.837)	**0.000**	2.266 (1.239-4.143)	**0.008**
Vascular invasion	2.983 (1.639-5.429)	**0.000**	1.433 (0.811-2.531)	0.215
High PFKFB4 expression	2.712 (1.697-4.333)	**0.000**	3.439 (2.111-5.601)	**0.000**

## Data Availability

The datasets used and analyzed during the current study are available from the corresponding author on reasonable request.

## References

[1] Ferlay J., Soerjomataram I., Dikshit R. (2015). Cancer incidence and mortality worldwide: sources, methods and major patterns in GLOBOCAN 2012. *International Journal of Cancer*.

[2] Torre L. A., Bray F., Siegel R. L., Ferlay J., Lortet-Tieulent J., Jemal A. (2015). Global cancer statistics, 2012. *CA: a Cancer Journal for Clinicians*.

[3] Dent R., Trudeau M., Pritchard K. I. (2007). Triple-negative breast cancer: clinical features and patterns of recurrence. *Clinical Cancer Research*.

[4] Foulkes W. D., Smith I. E., Reis-Filho J. S. (2010). Triple-negative breast cancer. *The New England Journal of Medicine*.

[5] Warburg O. (1956). On the origin of cancer cells. *Science*.

[6] Hockel M., Vaupel P. (2001). Tumor hypoxia: definitions and current clinical, biologic, and molecular aspects. *Journal of the National Cancer Institute*.

[7] The Cancer Genome Atlas Network (2012). Comprehensive molecular portraits of human breast tumours. *Nature*.

[8] Montagner M., Enzo E., Forcato M. (2012). SHARP1 suppresses breast cancer metastasis by promoting degradation of hypoxia-inducible factors. *Nature*.

[9] Lu H., Forbes R. A., Verma A. (2002). Hypoxia-inducible factor 1 activation by aerobic glycolysis implicates the Warburg effect in carcinogenesis. *The Journal of Biological Chemistry*.

[10] Hopfl G., Ogunshola O., Gassmann M. (2004). HIFs and tumors--causes and consequences. *American Journal of Physiology. Regulatory, Integrative and Comparative Physiology*.

[11] Stoeltzing O., McCarty M. F., Wey J. S. (2004). Role of hypoxia-inducible factor 1alpha in gastric cancer cell growth, angiogenesis, and vessel maturation. *Journal of the National Cancer Institute*.

[12] Okar D. A., Lange A. J. (1999). Fructose-2,6-bisphosphate and control of carbohydrate metabolism in eukaryotes. *BioFactors*.

[13] Kawaguchi T., Veech R. L., Uyeda K. (2001). Regulation of energy metabolism in macrophages during hypoxia:. *The Journal of Biological Chemistry*.

[14] Okar D. A., Lange A. J., Manzano À., Navarro-Sabatè A., Riera L.`., Bartrons R. (2001). PFK-2/FBPase-2: maker and breaker of the essential biofactor fructose-2,6-bisphosphate. *Trends in Biochemical Sciences*.

[15] Sakata J., Abe Y., Uyeda K. (1991). Molecular cloning of the DNA and expression and characterization of rat testes fructose-6-phosphate,2-kinase:fructose-2,6-bisphosphatase. *The Journal of Biological Chemistry*.

[16] Manzano A., Pérez J. X., Nadal M., Estivill X., Lange A., Bartrons R. (1999). Cloning, expression and chromosomal localization of a human testis 6-phosphofructo-2-kinase/fructose-2,6-bisphosphatase gene. *Gene*.

[17] Sakakibara R., Okudaira T., Fujiwara K. (1999). Tissue distribution of placenta-type 6-phosphofructo- 2-kinase/fructose-2,6-bisphosphatase. *Biochemical and Biophysical Research Communications*.

[18] Minchenko O. H., Ochiai A., Opentanova I. L. (2005). Overexpression of 6-phosphofructo-2-kinase/fructose-2,6-bisphosphatase-4 in the human breast and colon malignant tumors. *Biochimie*.

[19] Minchenko O. H., Opentanova I. L., Ogura T. (2005). Expression and hypoxia-responsiveness of 6-phosphofructo-2-kinase/fructose-2,6-bisphosphatase 4 in mammary gland malignant cell lines. *Acta Biochimica Polonica*.

[20] Yalcin A., Telang S., Clem B., Chesney J. (2009). Regulation of glucose metabolism by 6-phosphofructo-2-kinase/fructose-2,6-bisphosphatases in cancer. *Experimental and Molecular Pathology*.

[21] Pilkis S. J., Claus T. H., Kurland I. J., Lange A. J. (1995). 6-Phosphofructo-2-kinase/fructose-2,6-bisphosphatase: a metabolic signaling enzyme. *Annual Review of Biochemistry*.

[22] Minchenko O. H., Ogura T., Opentanova I. L. (2005). 6-Phosphofructo-2-kinase/fructose-2,6-bisphosphatase gene family overexpression in human lung tumor. *Ukr Biokhim Zh (1999)*.

[23] Minchenko O., Opentanova I., Caro J. (2003). Hypoxic regulation of the 6-phosphofructo-2-kinase/fructose-2,6-bisphosphatase gene family (PFKFB-1-4) expression in vivo. *FEBS Letters*.

[24] Chesney J., Clark J., Klarer A. C., Imbert-Fernandez Y., Lane A. N., Telang S. (2014). Fructose-2,6-bisphosphate synthesis by 6-phosphofructo-2-kinase/fructose-2,6-bisphosphatase 4 (PFKFB4) is required for the glycolytic response to hypoxia and tumor growth. *Oncotarget*.

[25] Ros S., Santos C. R., Moco S. (2012). Functional metabolic screen identifies 6-phosphofructo-2-kinase/fructose-2,6-biphosphatase 4 as an important regulator of prostate cancer cell survival. *Cancer Discovery*.

[26] Goidts V., Bageritz J., Puccio L. (2012). RNAi screening in glioma stem-like cells identifies PFKFB4 as a key molecule important for cancer cell survival. *Oncogene*.

[27] Kampf C., Olsson I. M., Ryberg U., Sjöstedt E., Pontén F. (2012). Production of tissue microarrays, immunohistochemistry staining and digitalization within the human protein atlas. *Journal of Visualized Experiments*.

[28] Miyai K., Iwaya K., Asano T., Tamai S., Matsubara O., Tsuda H. (2014). Fatty acid synthase overexpression in adult testicular germ cell tumors: potential role in the progression of non-seminomatous germ cell tumors. *Virchows Archiv*.

[29] Yi M., Ban Y., Tan Y., Xiong W., Li G., Xiang B. (2019). 6-Phosphofructo-2-kinase/fructose-2,6-biphosphatase 3 and 4: a pair of valves for fine-tuning of glucose metabolism in human cancer. *Mol Metab*.

[30] Semenza G. L. (1998). Hypoxia-inducible factor 1 and the molecular physiology of oxygen homeostasis. *The Journal of Laboratory and Clinical Medicine*.

[31] Ratcliffe P. J. (2002). From erythropoietin to oxygen: hypoxia-inducible factor hydroxylases and the hypoxia signal pathway. *Blood Purification*.

[32] Stolze I. P., Tian Y. M., Appelhoff R. J. (2004). Genetic analysis of the role of the asparaginyl hydroxylase factor inhibiting hypoxia-inducible factor (HIF) in regulating HIF transcriptional target genes. *The Journal of Biological Chemistry*.

[33] Taylor C., Mannion D., Miranda F. (2017). Loss of PFKFB4 induces cell death in mitotically arrested ovarian cancer cells. *Oncotarget*.

[34] Pegoraro C., Figueiredo A. L., Maczkowiak F., Pouponnot C., Eychène A., Monsoro-Burq A. H. (2015). PFKFB4 controls embryonic patterning via Akt signalling independently of glycolysis. *Nature Communications*.

[35] Gao R., Li D., Xun J. (2018). CD44ICD promotes breast cancer stemness via PFKFB4-mediated glucose metabolism. *Theranostics*.

[36] Gao R., Liu Y., Li D. (2018). PFKFB4 promotes breast cancer metastasis via induction of hyaluronan production in a p38-dependent manner. *Cellular Physiology and Biochemistry*.

[37] Dasgupta S., Rajapakshe K., Zhu B. (2018). Metabolic enzyme PFKFB4 activates transcriptional coactivator SRC-3 to drive breast cancer. *Nature*.

[38] Massague J. (2004). G1 cell-cycle control and cancer. *Nature*.

[39] Mayhew C. N., Perkin L. M., Zhang X., Sage J., Jacks T., Knudsen E. S. (2004). Discrete signaling pathways participate in RB-dependent responses to chemotherapeutic agents. *Oncogene*.

[40] Gong P., Wang Y., Liu G., Zhang J., Wang Z. (2013). New insight into Ki67 expression at the invasive front in breast cancer. *PLoS One*.

[41] Ros S., Flöter J., Kaymak I. (2017). 6-Phosphofructo-2-kinase/fructose-2,6-biphosphatase 4 is essential for p53-null cancer cells. *Oncogene*.

[42] Minchenko O. H., Ogura T., Opentanova I. L., Minchenko D. O., Esumi H. (2005). Splice isoform of 6-phosphofructo-2-kinase/fructose-2,6-bisphosphatase-4: expression and hypoxic regulation. *Molecular and Cellular Biochemistry*.

